# Supplementary material for: Murine implantation chamber formation precedes natural and artificial decidualization
Source: bioRxiv. 2025 Dec 31:2025.12.30.697086. Preprint. [Version 1] doi: 10.64898/2025.12.30.697086 (PMC12776327; doi:10.64898/2025.12.30.697086)
Supplement: 1 [file NIHPP2025.12.30.697086V1-supplement-1.pdf]

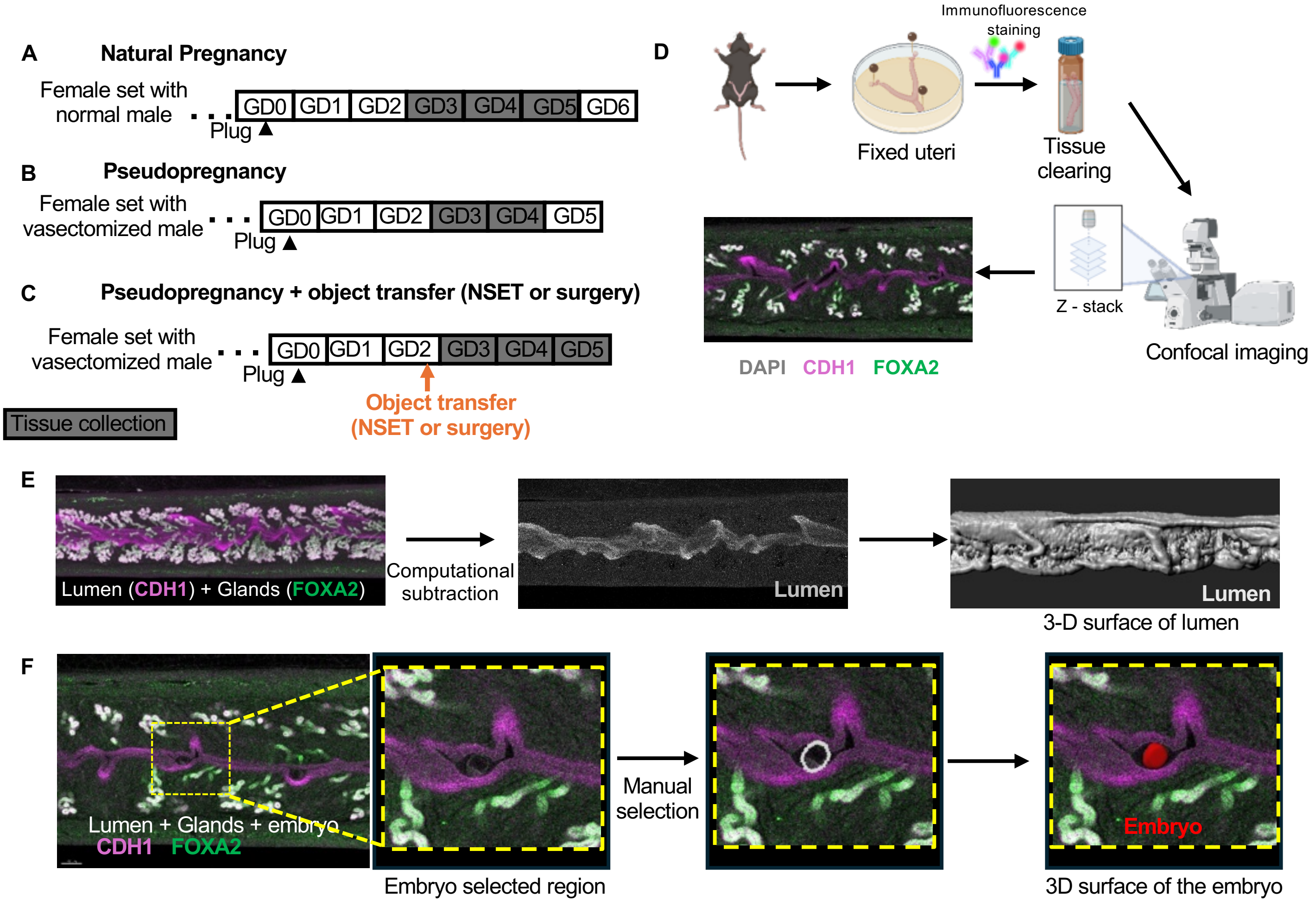

Supplementary Figure 1. Mouse models and methods used to determine implantation chamber formation is essential for decidualization (A-C) Schematic of mouse model in a natural pregnancy **(A)**, pseudopregnancy **(B)** and pseudopregnancy + object transfer using NSET or surgery. **(D)** Illustration of tissue fixation, whole mount immunofluorescence, and imaging protocol. **(E-F)** Process of 3D reconstruction of the lumen **(E)** and objects within the lumen **(F)** using Imaris v9.2.1. Arrowhead is GD0.5 when the plug was identified. Orange arrow indicates the NSET or surgery time. NSET – Non-surgical Embryo Transfer method.

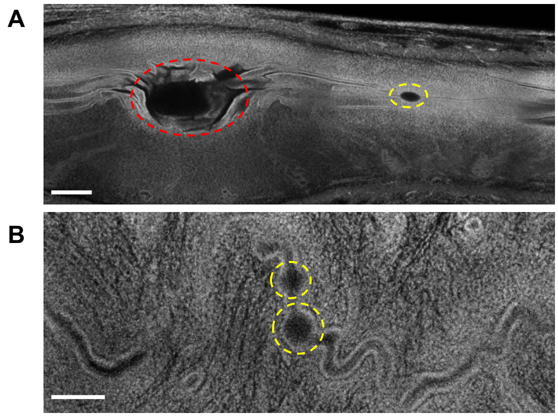

Supplementary Figure 2. Oil droplets and ConA-beads do not always form implantation chambers. (A-B) Representative confocal images showing variable chamber formation at GD4.5: a large oil droplet (red circle) initiates chamber formation, whereas a small oil droplet (yellow circle) does not form a chamber **(A),** two ConA-beads adjacent to each other (yellow circle) that failed to form a chamber **(B)**. Scale bars: **A:** 400 μm, **B:** 100 μm.

Supplementary Figure 2

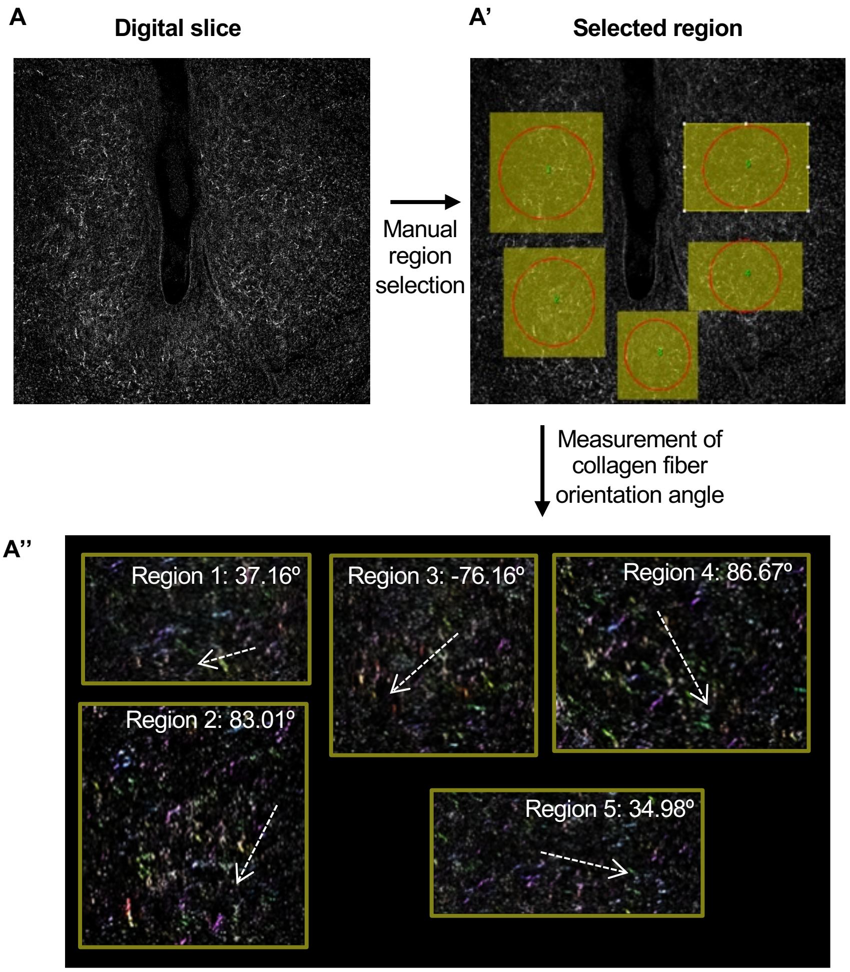

Supplementary Figure 3. Method for determining fiber orientation from SHG images using the OrientationJ plugin in ImageJ. **(A-A’’)** Pipeline for quantifying collagen fiber angles using OrientationJ involves the following steps: uploading SHG images and converting them to 8-bit format **(A)**, manually selecting five regions surrounding the chamber **(A’)**, and measuring the average angle of collagen fibers in the selected regions **(A’’)**. White arrows indicate the direction of collagen fibers in each region, SHG – Second Harmonic Generation.

Supplementary Figure 3
